# Artificial intelligence in prostate biopsy: diagnostic applications, risk stratification, and precision oncology

**DOI:** 10.3389/fonc.2026.1915581

**Published:** 2026-07-28

**Authors:** Nasar Alwahaibi

**Affiliations:** Department of Biomedical Science, College of Medicine and Health Sciences, Sultan Qaboos University, Muscat, Oman

**Keywords:** artificial intelligence, Gleason grading, prostate biopsy, prostate cancer, risk stratification

## Abstract

Prostate biopsy remains the cornerstone for the diagnosis, risk stratification, and management of prostate cancer. However, biopsy interpretation is challenged by sampling limitations, tumor heterogeneity, and interobserver variability in Gleason grading. Recent advances in digital pathology and artificial intelligence (AI) have created new opportunities to improve diagnostic accuracy, reproducibility, and clinical decision-making. This narrative mini-review summarizes current evidence regarding AI applications in prostate biopsy evaluation. Relevant studies published between 2015 and 2026 were reviewed, focusing on AI-assisted cancer detection, automated Gleason grading, quantitative pathology, prognostic assessment, and multimodal approaches integrating histopathological, molecular, and clinical data. AI-based systems have demonstrated high accuracy in prostate cancer detection, Gleason pattern classification, and tumor burden assessment, with several studies showing strong concordance with expert genitourinary pathologists. These technologies have the potential to improve diagnostic consistency, enhance workflow efficiency, refine risk stratification, and support treatment planning. Emerging multimodal AI models integrating histopathological, genomic, imaging, and clinical information may further improve prognostic assessment and facilitate precision oncology approaches. However, challenges remain, including limited prospective validation, data heterogeneity, regulatory considerations, model interpretability, and integration into routine clinical workflows. AI is emerging as a valuable adjunct in prostate biopsy evaluation, with the potential to enhance diagnostic precision, grading reproducibility, and personalized patient management. Continued multicenter validation, development of explainable AI frameworks, and effective integration into multidisciplinary prostate cancer care pathways will be essential for successful clinical adoption and improved patient outcomes.

## Introduction

Prostate cancer remains one of the most frequently diagnosed malignancies among men worldwide and is a leading cause of cancer-related morbidity and mortality (1). Accurate diagnosis is fundamental to optimal patient management, influencing decisions regarding active surveillance, focal therapy, radical treatment, and long-term follow-up. Histopathological evaluation of prostate needle biopsies remains the cornerstone of diagnosis, providing critical information on tumor presence, grade, and extent that guides risk stratification, prognostic assessment, and therapeutic planning (2). The Gleason grading system, subsequently refined by the International Society of Urological Pathology (ISUP), continues to serve as the principal framework for assessing tumor aggressiveness and predicting clinical outcomes (3).

Despite standardized diagnostic criteria, prostate biopsy interpretation remains challenging. Limited tissue sampling, tumor heterogeneity, and the presence of benign lesions that mimic carcinoma can complicate diagnosis and grading, particularly in small biopsy cores (4, 5). Furthermore, interobserver variability in Gleason grading persists even among experienced pathologists, potentially affecting risk classification, treatment selection, and patient outcomes (6). As prostate cancer management increasingly shifts toward precision oncology, there is a growing need for tools that enhance diagnostic accuracy, reproducibility, and efficiency while supporting personalized clinical decision-making.

Recent advances in digital pathology and artificial intelligence (AI) have generated considerable interest in prostate cancer diagnostics. AI-based systems can analyze whole-slide images to detect cancer, classify Gleason patterns, quantify tumor burden, and identify histomorphological features associated with prognosis and disease progression (7). By providing objective and reproducible assessments, these technologies have the potential to augment pathologist performance, improve diagnostic consistency, and support risk-adapted patient management. Over the past decade, multiple studies have demonstrated the feasibility and high accuracy of AI-assisted prostate biopsy analysis, with several platforms advancing toward clinical validation and real-world implementation (8–10).

This review summarizes current and emerging applications of AI in prostate biopsy evaluation, with a particular focus on cancer detection, Gleason grading, quantitative pathology, and risk stratification. We also discuss the clinical implications of these technologies, ongoing barriers to implementation, and future directions for integrating AI into precision prostate cancer diagnosis and management.

## Methods

A narrative review was conducted to summarize current evidence on the application of AI in prostate biopsy evaluation and its implications for prostate cancer diagnosis and management. Relevant literature published between January 2015 and March 2026 was identified through searches of PubMed/MEDLINE, Scopus, and Web of Science using combinations of the terms “prostate cancer, “ “prostate biopsy, “ “artificial intelligence, “ “machine learning, “ “deep learning, “ “digital pathology, “ and “Gleason grading”.

Studies were selected based on their relevance to AI-assisted histopathological assessment of prostate biopsies, including cancer detection, Gleason grading, quantitative pathology, prognostic assessment, and clinical implementation. The identified literature was reviewed and synthesized narratively with emphasis on diagnostic performance, clinical utility, and future applications. Owing to the heterogeneity of study designs, datasets, and AI methodologies, a formal systematic review or meta-analysis was not performed.

### Diagnostic challenges in prostate biopsy

Prostate needle biopsy remains the cornerstone for diagnosing prostate cancer and determining disease risk; however, several diagnostic challenges can affect the accuracy, reproducibility, and clinical utility of biopsy interpretation (11). Because biopsies sample only a small proportion of the prostate gland, clinically significant tumors may be missed or underrepresented, potentially leading to underestimation of disease burden and inaccurate risk classification (12). In addition, intratumoral heterogeneity is common, with multiple Gleason patterns frequently present within the same lesion, complicating accurate grading and prognostic assessment (13). As treatment decisions, including active surveillance, focal therapy, radical prostatectomy, radiotherapy, and systemic treatment, are closely linked to biopsy findings, precise histopathological evaluation is critical for optimal patient management.

Another challenge is distinguishing prostate cancer from benign conditions that can mimic malignancy, including atypical adenomatous hyperplasia, atrophy, and basal cell hyperplasia (14, 15). Although immunohistochemical markers such as p63, high-molecular-weight cytokeratin, and α-methylacyl-CoA racemase (AMACR) can assist in diagnostically challenging cases, interpretation may remain difficult in limited, fragmented, or equivocal biopsy samples (16, 17). Furthermore, interobserver variability remains a recognized limitation, particularly in differentiating Gleason pattern 3 from pattern 4, a distinction with important implications for risk stratification, prognosis, and treatment selection (18). Collectively, these challenges highlight the need for more objective and standardized diagnostic approaches and have stimulated growing interest in AI-based tools capable of providing reproducible, quantitative, and clinically actionable assessments of prostate biopsy specimens (19).

### Artificial intelligence for prostate cancer detection

AI has emerged as one of the most promising technologies for improving prostate cancer detection in biopsy specimens. Most contemporary systems utilize deep learning approaches, particularly convolutional neural networks (CNNs), to analyze digitized whole-slide images and identify histological features associated with malignancy (20). Trained on large datasets of annotated prostate biopsies, these models can distinguish benign from malignant glands and generate probability maps that highlight suspicious regions, thereby supporting pathologists during diagnostic evaluation.

Several studies have reported diagnostic performance comparable to that of expert genitourinary pathologists, with high sensitivity and specificity for cancer detection (21, 22). Beyond diagnostic accuracy, AI-assisted detection may provide important clinical benefits by reducing oversight of small tumor foci, improving diagnostic reproducibility, and facilitating more efficient review of increasing biopsy workloads. Early and accurate identification of clinically significant prostate cancer is particularly important for risk stratification and treatment planning, as diagnostic findings directly influence decisions regarding active surveillance, focal therapy, and definitive treatment.

**Figure 1:**
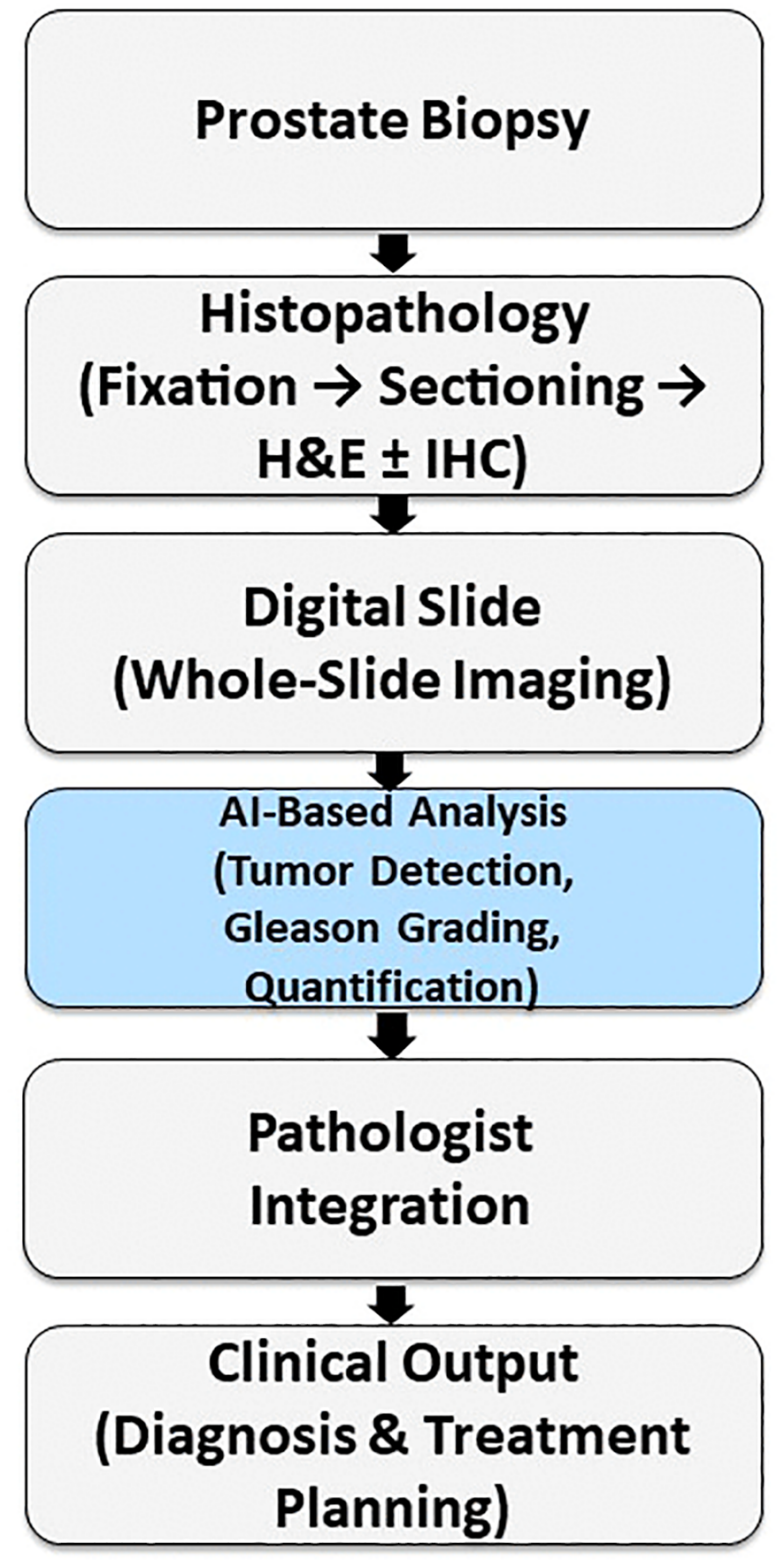
Artificial intelligence -assisted workflow for prostate biopsy diagnostics.

Consequently, AI is increasingly being investigated as a screening, triage, and decision-support tool capable of prioritizing suspicious cases and directing pathologists to diagnostically relevant regions of interest (23, 24). These applications have the potential to improve workflow efficiency while maintaining diagnostic quality, particularly in high-volume pathology laboratories. As digital pathology adoption continues to expand, AI-assisted cancer detection is expected to become an increasingly important component of precision diagnostic workflows in prostate cancer. An overview of the AI-assisted workflow in prostate biopsy diagnostics is illustrated in **Figure 1**.

### Automated Gleason grading

Accurate Gleason grading remains one of the most important determinants of prostate cancer risk stratification, prognosis, and treatment selection. Distinguishing between Gleason patterns, particularly patterns 3 and 4, can be challenging and is subject to interobserver variability, potentially influencing eligibility for active surveillance, focal therapy, and definitive treatment (25, 26). To address these challenges, AI algorithms have been developed to perform automated Gleason grading using digitized biopsy slides. Most systems employ deep learning models capable of analyzing glandular architecture and classifying tumors according to established grading criteria (27, 28).

Several validation studies have demonstrated strong agreement between AI-assisted grading systems and expert genitourinary pathologists, suggesting that these tools may improve grading consistency, reproducibility, and diagnostic standardization (29–31). By providing objective assessments of tumor architecture, AI has the potential to reduce diagnostic variability and support more uniform risk classification across institutions (32). Such improvements may be particularly valuable in settings where subspecialty genitourinary pathology expertise is limited.

Beyond conventional grading, AI-derived quantitative features may capture subtle morphological characteristics associated with tumor aggressiveness, biological behavior, and clinical outcomes, providing additional prognostic information beyond traditional histopathological assessment (33). As computational pathology continues to evolve, automated Gleason grading is likely to become an important component of precision oncology workflows, supporting risk-adapted management strategies and more individualized treatment decision-making in patients with prostate cancer.

### Quantitative pathology and tumor burden assessment

Assessment of tumor burden is a critical component of prostate biopsy reporting and plays an important role in risk stratification, treatment selection, and eligibility for active surveillance (34, 35). Parameters such as the percentage of tumor involvement within biopsy cores, the number of positive cores, and the extent of high-grade disease are routinely incorporated into clinical decision-making. Traditionally, these measurements are estimated visually, introducing the potential for interobserver variability and inconsistencies in reporting.

AI-based image analysis platforms can automatically quantify tumor area, gland density, and architectural features across whole-slide images, providing more standardized, objective, and reproducible assessments of tumor burden (36). Beyond routine measurements, computational pathology approaches can extract large numbers of quantitative morphological features that are not readily discernible through conventional microscopic evaluation. These digital biomarkers may improve prognostic assessment, refine risk stratification, and identify patients at increased risk of disease progression, treatment failure, or adverse clinical outcomes (37). By enabling more precise characterization of tumor biology, quantitative pathology has the potential to support individualized management strategies and facilitate risk-adapted treatment approaches in prostate cancer.

### Multimodal integration and precision oncology

The growing availability of genomic, molecular, and clinical data has accelerated interest in integrating diverse sources of information to improve prostate cancer management. Given the substantial biological heterogeneity of prostate cancer, conventional histopathological assessment alone may not fully capture the complexity of disease behavior or treatment response (38). AI provides a powerful framework for integrating histopathological, molecular, imaging, and clinical data, enabling the identification of complex patterns that may enhance prognostic accuracy and therapeutic decision-making.

Recent studies have demonstrated that AI models can predict specific molecular alterations and genomic signatures directly from histopathological images, suggesting that tissue morphology may reflect underlying biological pathways and tumor behavior (39, 40). More recently, multimodal AI frameworks have extended this approach by integrating biopsy histopathology with liquid biopsy biomarkers, multiparametric magnetic resonance imaging (mpMRI), genomic profiling, and clinical metadata, such as prostate-specific antigen (PSA) levels, age, and other clinicopathological variables. Such synergistic data fusion has the potential to improve early cancer detection, refine risk stratification, predict treatment response, and support personalized therapeutic decision-making, representing an important step toward precision oncology. However, further prospective validation, standardized data harmonization, and the development of interpretable AI models are required before these multimodal approaches can be routinely implemented in clinical practice (41).

The integration of digital pathology with molecular profiling, imaging findings, and clinical variables may facilitate more accurate risk stratification and support personalized treatment approaches. Such multimodal models have the potential to identify patients most likely to benefit from targeted therapies, treatment intensification, or active surveillance, thereby advancing the goals of precision oncology and improving patient-centered care in prostate cancer management.

### Summary of AI applications in prostate biopsy

The studies summarized in **Table 1** demonstrate the rapidly expanding role of AI in prostate biopsy evaluation, particularly in cancer detection, Gleason grading, tumor burden assessment, and workflow optimization. Across multiple studies, AI systems have achieved high diagnostic accuracy and strong concordance with expert pathologists, supporting their potential as adjunctive tools for improving diagnostic consistency, reducing interobserver variability, and enhancing risk stratification (42–45). These capabilities are particularly important in prostate cancer, where biopsy findings directly influence prognosis, treatment selection, and eligibility for active surveillance.

**Table 1:** Key artificial intelligence studies in prostate biopsy and digital pathology.

AI model	Model type	Application	Dataset	Key performance	Key contribution	Limitations	Maturity level	Clinical impact	Ref
Integrated AI pipeline (A!MagQC, A!HistoClouds, PAI)	CNN-based pipeline + workflow integration	Gleason grading	Multi-scanner WSI datasets	F1: 0.80; QWK: 0.71 (increased to 0.88 cross-scanner)	Cross-scanner generalization; improved efficiency; pathologist–AI interaction	Requires continuous data integration; scanner variability	Translational/Near-clinical	Standardization of Gleason grading; improved workflow efficiency	(41)
ResNet multi-scale deep learning	CNN (ResNet)	Cancer grading	Diagset dataset	Accuracy: 0.999	High-accuracy patch-level grading	Risk of overfitting; dataset dependency	Experimental	Patch-level grading support	(42)
CNN + U-Net + Aquila optimizer	CNN + segmentation (U-Net) + optimization algorithm	Classification & segmentation	Multi-dataset	Accuracy: up to 100%; AUC: 0.9778	Hybrid classification–segmentation framework	Overfitting; dataset variability	Experimental/Preclinical	Automated tumor detection and localization	(43)
CNN + GLCM + wavelet features	Hybrid (deep learning + handcrafted features)	Histological grading	Pathology datasets	Accuracy: 97.3%; AUC: 0.95	Combined texture + deep features improve grading	Needs multi-center validation	Experimental	Improved diagnostic accuracy	(44)
Federated attention-consistent learning (FACL)	Federated learning + attention-based deep learning	Diagnosis & Gleason grading	19, 461 WSIs (multi-center)	AUC: 0.9718; Kappa: 0.8463	Privacy-preserving multi-center training; improved generalization	Infrastructure complexity	Advanced Translational	Scalable AI deployment across institutions	(45)
Pixel-wise AI + virtual biopsy	Segmentation (pixel-wise CNN) + simulation model	Tumor detection & biopsy optimization	442 slides; 115 RP cases	Sensitivity: 0.99; Specificity: 0.90	Virtual biopsy simulation; optimization of biopsy strategy	Requires high-quality annotations	Translational/Preclinical	Improved biopsy sampling strategy	(46)
Swin Transformer + super-resolution (SwinIR)	Transformer + image super-resolution	Gleason grading (MPM imaging)	Multiphoton microscopy images	Accuracy: ~90%	Real-time grading with enhanced image quality	Requires specialized imaging	Early Translational	Potential intra-procedural grading	(47)
CNN + stimulated Raman histology (SRH)	CNN + real-time imaging integration	Real-time biopsy diagnosis	303 training/113 test biopsies	Accuracy: 96.5%; Sensitivity: 96.3%	Near real-time diagnosis without tissue processing	Limited SRH availability	High Translational/Near-clinical	Rapid intraoperative diagnosis	(48)

AI, artificial intelligence; WSIs, whole-slide images; F1, F1 score; QWK, quadratic weighted kappa; CNN, convolutional neural network; U-Net (convolutional neural network architecture for image segmentation); AUC, area under the receiver operating characteristic curve; GG, Gleason grading; FACL, federated attention-consistent learning; RP, radical prostatectomy; MPM, multiphoton microscopy; SRH, stimulated Raman histology; GLCM, gray-level co-occurrence matrix; ResNet, residual neural network; SwinIR, Swin Transformer for Image Restoration; Swin Transformer (transformer-based deep learning architecture using shifted windows); MobileNet (lightweight convolutional neural network architecture).

Beyond improving diagnostic performance, AI has shown considerable promise in enhancing workflow efficiency. Several studies have demonstrated that AI-assisted platforms can identify suspicious regions, reduce slide review times, and facilitate more standardized reporting while maintaining high diagnostic accuracy (42, 43). Such improvements may help address increasing pathology workloads associated with the growing demand for prostate cancer diagnosis and management.

Recent advances have focused on improving the scalability, robustness, and clinical applicability of AI systems. Multi-institutional validation studies, cross-platform approaches, and federated learning frameworks have sought to overcome challenges related to data heterogeneity, model generalizability, and patient privacy (46). In parallel, emerging technologies integrating AI with advanced imaging modalities have demonstrated the potential for rapid or near real-time biopsy assessment, potentially reducing diagnostic turnaround times and supporting more timely clinical decision-making (47–49).

Despite these encouraging developments, most AI applications remain in the validation or early implementation phase. Many studies are based on retrospective datasets and require further prospective, multicenter evaluation before widespread clinical adoption. Additional challenges include regulatory approval, interoperability across digital pathology platforms, workflow integration, and clinician acceptance. At present, AI should be viewed as a decision-support technology that augments rather than replaces expert pathological assessment. Nevertheless, the growing body of evidence suggests that AI has the potential to improve diagnostic standardization, refine prognostic assessment, and support more personalized and precision-based management of patients with prostate cancer.

### Challenges and limitations

Despite substantial advances in AI-assisted prostate biopsy analysis, several challenges continue to limit widespread clinical implementation. Although some AI systems have achieved diagnostic performance comparable to expert pathologists, considerable variability exists among published studies with respect to datasets, algorithms, validation strategies, and performance metrics, making direct comparison and clinical translation difficult (50–52). In addition, many models have been developed and validated using retrospective, single-center cohorts, raising concerns regarding their generalizability across diverse patient populations, healthcare systems, and imaging platforms (53). Robust prospective and multicenter validation studies remain essential before these technologies can be routinely incorporated into clinical practice.

Practical barriers also remain significant. Successful implementation of AI in routine pathology workflows requires substantial investment in digital pathology infrastructure, image storage systems, computational resources, and personnel training (54). Furthermore, regulatory approval remains a major challenge. AI-based diagnostic systems intended for clinical use must comply with regulatory frameworks such as the U.S. Food and Drug Administration (FDA) requirements and the European Union Medical Device Regulation (EU MDR), which require rigorous evidence of analytical validity, clinical performance, safety, and continuous post-market monitoring (55, 56). The limited availability of large, well-annotated reference datasets further complicates independent validation, benchmarking, and external reproducibility of AI systems (57, 58).

Another important challenge relates to clinician confidence and acceptance. Many deep learning models operate as “black-box” systems, providing limited insight into the features underlying diagnostic predictions (59, 60). This lack of interpretability may hinder trust among pathologists and clinicians, particularly when AI outputs influence risk stratification, prognostic assessment, and treatment decisions. In addition, underrepresentation of certain populations in training datasets may introduce algorithmic bias, potentially reducing model performance in specific patient groups and exacerbating healthcare disparities if not adequately addressed (61, 62). Addressing these challenges will be critical to ensuring that AI technologies are reliable, equitable, and clinically applicable across diverse healthcare settings.

### Future directions

Future developments in AI-assisted prostate cancer diagnostics are likely to focus on improving clinical applicability, generalizability, and seamless integration into diagnostic and therapeutic workflows (63). Large multicenter collaborations and federated learning frameworks may facilitate the development of more robust and representative models while preserving patient privacy and data security (64). At the same time, advances in explainable AI are expected to improve model transparency by enabling clinicians to better understand the histological features driving algorithm predictions, thereby increasing trust, supporting regulatory acceptance, and facilitating clinical implementation (65, 66).

Beyond conventional histopathology, AI is increasingly being integrated with molecular, genomic, and spatially resolved technologies. Combining histomorphological assessment with spatial transcriptomics, proteomics, and other omics platforms may provide deeper insights into tumor heterogeneity, biological behavior, therapeutic response, and mechanisms of disease progression, thereby supporting more precise risk stratification and personalized treatment selection (67–70). Similarly, AI-assisted analysis of immunohistochemical biomarkers may enable more objective and reproducible quantification of biomarker expression, improving diagnostic accuracy, prognostic assessment, and patient selection for targeted therapeutic strategies in selected clinical settings (71).

Future advances are also likely to support the development of integrated multimodal models that combine histopathological, molecular, radiological, and clinical data to generate comprehensive patient-specific risk profiles. Such approaches may further refine prognostic prediction and facilitate precision oncology by identifying patients most likely to benefit from active surveillance, treatment intensification, or targeted therapies.

Ultimately, the future of AI in prostate cancer diagnosis is likely to depend on effective human–AI collaboration rather than full automation. In this paradigm, AI serves as a decision-support tool that enhances diagnostic consistency, efficiency, quantitative assessment, and risk stratification, while final clinical and pathological decisions remain under expert supervision (72). Such collaborative approaches are likely to maximize the benefits of AI while preserving the clinical judgment essential for individualized patient care.

## Conclusion

AI is emerging as a valuable adjunct in prostate biopsy evaluation, with the potential to improve cancer detection, enhance Gleason grading consistency, and provide quantitative biomarkers that support risk stratification, prognostic assessment, and treatment planning. Although important challenges remain, including the need for prospective validation, regulatory oversight, and integration into routine clinical workflows, current evidence suggests that AI can improve diagnostic accuracy, reproducibility, and efficiency when used alongside expert pathological assessment. As digital pathology adoption expands and multimodal AI approaches incorporating molecular, imaging, and clinical data continue to evolve, AI-assisted biopsy evaluation is expected to play an increasingly important role in precision oncology and personalized prostate cancer care. Future progress will depend on robust multicenter validation studies, demonstration of clinical utility, and successful integration into multidisciplinary prostate cancer management pathways to ensure meaningful improvements in patient outcomes.
